# Use of multiple‐locus variable‐number of tandem repeats analysis (MLVA) to investigate genetic diversity of *Salmonella enterica* subsp. *enterica* serovar Typhimurium isolates from human, food, and veterinary sources

**DOI:** 10.1002/mbo3.528

**Published:** 2017-08-23

**Authors:** Gergana Mateva, Karl Pedersen, Gitte Sørensen, Galina Asseva, Hristo Daskalov, Petar Petrov, Todor Kantardjiev, Irina Alexandar, Charlotta Löfström

**Affiliations:** ^1^ National Diagnostic Research Veterinary Institute Sofia Bulgaria; ^2^ National Veterinary Institute Technical University of Denmark Frederiksberg C Denmark; ^3^ National Food Institute Technical University of Denmark Søborg Denmark; ^4^ National Center of Infectious and Parasitic Diseases Sofia Bulgaria; ^5^ Institute of Molecular Biology Bulgarian Academy of Sciences Sofia Bulgaria; ^6^ Agrifood and Bioscience RISE Research Institutes of Sweden Lund Sweden

**Keywords:** antimicrobial resistance, laboratory surveillance, MLVA, public health, *Salmonella* genetic diversity, zoonoses

## Abstract

*Salmonella enterica* subspecies *enterica* serovar Typhimurium is the most common zoonotic pathogen in Bulgaria. To allow efficient outbreak investigations and surveillance in the food chain, accurate and discriminatory methods for typing are needed. This study evaluated the use of multiple‐locus variable‐number of tandem repeats analysis (MLVA) and compared results with antimicrobial resistance (AMR) determinations for 100 *S*. Typhimurium strains isolated in Bulgaria during 2008–2012 (50 veterinary/food and 50 human isolates). Results showed that isolates were divided into 80 and 34 groups using MLVA and AMR, respectively. Simpson's index of diversity was determined to 0.994 ± 0.003 and 0.945 ± 0.012. The most frequently encountered MLVA profiles were 3‐11‐9‐NA‐211 (*n* = 5); 3‐12‐9‐NA‐211 (*n* = 3); 3‐12‐11‐21‐311 (*n* = 3); 3‐17‐10‐NA‐311 (*n* = 3); 2‐20‐9‐7‐212 (*n* = 3); and 2‐23‐NA‐NA‐111 (*n* = 3). No clustering of isolates related to susceptibility/resistance to antimicrobials, source of isolation, or year of isolation was observed. Some MLVA types were found in both human and veterinary/food isolates, indicating a possible route of transmission. A majority (83%) of the isolates were found to be resistant against at least one antimicrobial and 44% against ≥4 antimicrobials. Further studies are needed to verify MLVA usefulness over a longer period of time and with more isolates, including outbreak strains.

## 1 INTRODUCTION

Nontyphoid salmonellae are among the leading causes for human food‐borne infections worldwide. Recent estimates made by the European Centre for Disease Prevention and Control (ECDC) and the European Food Safety Authority (EFSA) have stated that salmonellosis was the second most commonly reported zoonotic illness in Europe with 23.7 cases per 100,000 inhabitants (EFSA and ECDC, 2013; Lahuerta et al., 2011; Majowicz et al., 2010). Since 2000 the two most frequently reported serotypes in Bulgaria are *Salmonella enterica* subspecies *enterica* serovar Enteritidis (*S*. Enteritidis) and *Salmonella* Typhimurium (Anonymous, 2013; WHO 2014). Annually, a number of food‐borne outbreaks linked to the consumption of different food of animal origin occur among humans worldwide (Dyet, Turbitt, & Carter, 2011). Recent studies also describe the occurrence of *Salmonella* contamination in food of nonanimal origin, for example, beans (Sadler‐Reeves et al., 2016). To prevent human salmonellosis caused by the consumption of contaminated food, there is a need to develop modern diagnostic methods that allow efficient outbreak investigations and surveillance.

The currently existing phenotyping methods for *Salmonella*, including serotyping, phage typing, and antimicrobial resistance (AMR) determination, have several disadvantages, such as being time consuming and laborious, and having insufficient discriminatory power for certain purposes (Lindstedt, 2011; Wattiau, Boland, & Bertrand, 2011). Powerful genotyping methods, for example, multiple‐locus variable‐number of tandem repeats (VNTR) analysis (MLVA), pulsed‐field gel electrophoresis (PFGE), multilocus sequence typing (MLST), and more recently whole genome sequencing (WGS), have been introduced into laboratory practice to solve some of the problems with the existing classical protocols (Ronholm, Nasheri, Petronella, & Pagotto, 2016; Wattiau et al., 2011). However, successful implementation of these techniques requires specialized equipment and training of personnel, thus making it a challenge in laboratories with limited resources.

The objective of this study was to apply MLVA to investigate the genetic diversity of a selection of *S. *Typhimurium isolates from human, veterinary, and food sources in Bulgaria from 2006 to 2012 to obtain background information about MLVA types, and the discriminatory ability of the different MLVA loci, in order to evaluate its applicability for routine surveillance and outbreak investigations. The results from MLVA were compared with the AMR profiles (currently used typing method) obtained for the same isolates and factors such as typeability (ability to generate a typing result) and discriminatory power were assessed.

## 2 RESULTS AND DISCUSSION

**Figure 1:**
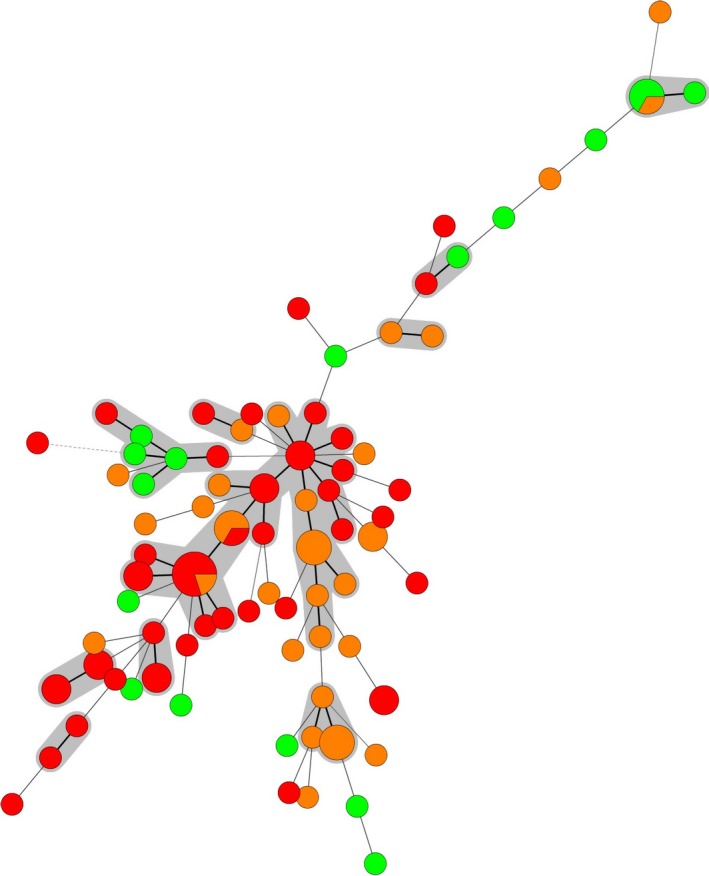
Minimum spanning tree of multiple‐locus variable‐number of tandem repeats analysis (MLVA) data for the 100 Bulgarian *Salmonella* serovar Typhimurium isolates. Colors represent antimicrobial resistance patterns (green: sensitive, orange: resistant to 1–3 antimicrobials, and red: multiresistant, i.e., resistant to >3 antimicrobials). Bold solid lines between circles indicate one locus difference, solid lines two loci difference, and dashed lines ≥3 loci difference between MLVA types. The diameter of the circle is proportional to the number of isolates in that particular type. The gray circles represent MLVA types with one loci difference

**Figure 2:**
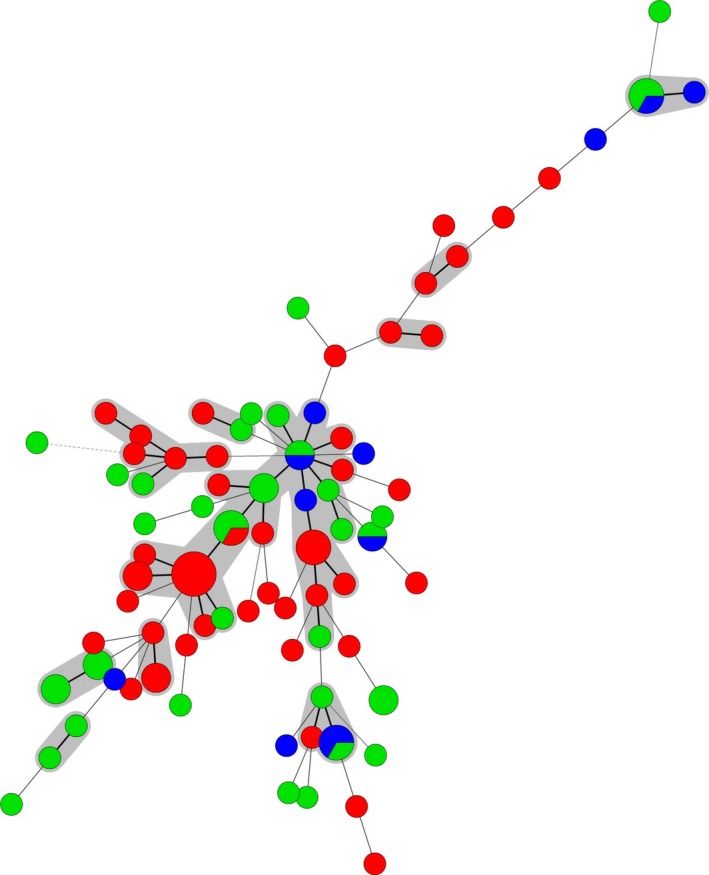
Minimum spanning tree of multiple‐locus variable‐number of tandem repeats analysis (MLVA) data for the 100 Bulgarian *Salmonella* serovar Typhimurium isolates. Colors represent source: red—human, green—food, and blue—veterinary. Bold solid lines between circles indicate one locus difference, solid lines two loci difference, and dashed lines ≥3 loci difference between MLVA types. The diameter of the circle is proportional to the number of isolates of that particular type. The gray circles represent MLVA types with one loci difference

**Figure 3:**
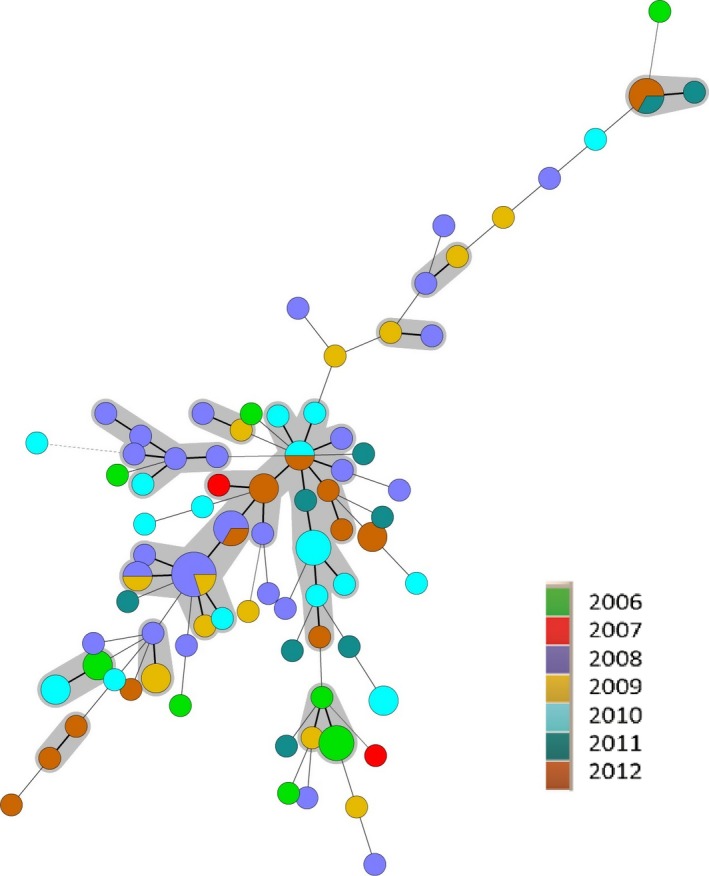
Minimum spanning tree of multiple‐locus variable‐number of tandem repeats analysis (MLVA) data for the 100 Bulgarian *Salmonella* serovar Typhimurium isolates. Colors represent year of isolation (2008–2012), see figure insert. Bold solid lines between circles indicate one locus difference, solid lines two loci difference, and dashed lines ≥3 loci difference between MLVA types. The diameter of the circle is proportional to the number of isolates in that particular type. The gray circles represent MLVA types with one loci difference

As *S*. Typhimurium is one of the serotypes that are most often isolated from humans, as well as animals and food (EFSA and ECDC, 2013), it is important from a public health perspective to rapidly differentiate between strains to enable efficient tracing throughout the food production chain. A high diversity among the 100 investigated *S. *Typhimurium isolates from Bulgaria was found with a total number of 80 different MLVA profiles (Table 1, Table S1). The most frequently encountered profiles were 3‐11‐9‐NA‐211 (*n* = 5); 3‐12‐9‐NA‐211 (*n* = 3); 3‐12‐11‐21‐311 (*n* = 3); 3‐17‐10‐NA‐311 (*n* = 3); 2‐20‐9‐7‐212 (*n* = 3); and 2‐23‐NA‐NA‐111 (*n* = 3). Nine of the MLVA profiles contained two isolates and 71% of the studied isolates had a unique MLVA profile. The relationship between the isolates was investigated using minimum spanning trees (MSTs) (Figures 1, 2, 3). No clustering of isolates related to susceptibility/resistance to antimicrobials (Figure 1), source of isolation (Figure 2), or year of isolation (Figure 3) was observed. Several of the MLVA types were found in isolates from both human and veterinary/food sources (Figure 2). When joining single locus variants (SLVs) into groups, the 80 MLVA types for the 100 Bulgarian isolates were merged into 60 groups.

**Table 1:** Comparison of antimicrobial resistance patterns (AMR) and multiple‐locus variable‐number of tandem repeats analysis (MLVA) results for the 100 Bulgarian *Salmonella* serovar Typhimurium isolates

Typing method	No. of groups	Frequent types (no. of isolates)^a^	Index of diversity	
			Hunter–Gaston′s ± standard deviation^b^	Shannon–Weiner′s^c^
AMR	34	Sensitive (17) ASSuT (11) ACSSuT (6) Su (6) ACSu (5) ACSSu (5) A (4) ACCbT (4) ACGSSuT(3) AST (3) AT (3) Cp (3)	0.945 ± 0.012	H’ = 3.1302 E = 0.8876
MLVA	80	3‐11‐9‐NA‐211 (5) 3‐12‐11‐21‐311 (3) 2‐20‐9‐7‐212 (3) 3‐12‐9‐NA‐211 (3) 3‐17‐10‐NA‐311 (3)	0.994 ± 0.003	H′ = 4.2820 E = 0.9772

a A, ampicillin; C, chloramphenicol; Cp, ciprofloxacin; G, gentamicin; S, streptomycin; Su, sulfonamides; T, tetracycline; Cb, carbenicillin. MLVA types given in the following format: STTR5, STTR9, STTR6, STTR10, and STTR3 according to (Larsson et al., 2009).

b Calculated as previously described (Hunter & Gaston, 1988) given as value ± standard deviation.

c Shannon–Weiner diversity index where H′ represents the subtype diversity, that is, the number of different subtypes, and E is a measure of evenness, that is, how evenly the subtypes are distributed in the population sampled.

MLVA‐typing has previously been used successfully for outbreak investigations (Bruun et al., 2009; Heck, 2009). This method has high discriminatory power and has been proposed as an alternative for genotyping of highly clonal groups of bacteria (Adhikari et al., 2010). MLVA has also been shown to produce reproducible results that easily can be shared among laboratories (Larsson, Torpdahl, & Nielsen, 2013). This is an important aspect to consider in international exchange of data, for example, within the EU. In this study, the MLVA method was found to discriminate well between the investigated *S*. Typhimurium isolates. It is therefore anticipated to be a promising tool in outbreak investigations and to achieve a fast diagnosis, compared to AMR determination which is, in combination with serotyping, the currently used typing method at the Bulgarian public health laboratories.

However, MLVA has been proposed to be unsuitable to determine clonality among the isolates spread over a longer time period (Tapalski, Hendriksen, Hasman, Ahrens, & Aarestrup, 2007). This technique therefore has to be used in combination with and supported by epidemiological data to provide useful information in an outbreak situation (Ramisse et al., 2004; Tapalski et al., 2007). In line with this, it has been shown that isolates with single locus variants of a MLVA type can belong to the same outbreak (Petersen et al., 2011), and that the *in vitro* and *in vivo* stability cannot be assumed to be the same for all *Salmonella* MLVA types (Barua et al., 2013; Wattiau et al., 2011). Similarly, isolates can lose or obtain AMR during the course of an outbreak (Nielsen, Torpdahl, Ethelberg, & Hammerum, 2009), meaning that also this kind of data has to be used with caution during outbreak investigations.

Several of the most commonly found MLVA types in our study (3‐11‐9‐NA‐21 and 3‐12‐9‐NA‐211) have also been found in other investigations, in some cases including the monophasic variant of *S*. Typhimurium, for example, in the UK (Hopkins et al., 2010), Belgium (Wuyts et al., 2013), Denmark (Arguello et al., 2014), and Sweden (SVA 2015). However, we also found some rare profiles that, to our knowledge, have not been isolated previously on a frequent basis. When comparing the MLVA types for the Bulgarian strains to neighboring countries, for example, Romania (Usein et al., 2010), no considerable overlap in types was noted. This indicates that transmission over the borders with *Salmonella* was not the major factor contributing to the Bulgarian MLVA type diversity. However, this conclusion has to be interpreted with caution, since different approaches for selecting strains were used in the different studies and also a limited number of strains were investigated. Moreover, differences in monitoring, methods for selection of isolates and laboratory methods could contribute to the differences. Due to the discontinuation of the MLVA‐Net database (Guigon, Cheval, Cahuzac, & Brisse, 2008) and the change in nomenclature for the MLVA *S*. Typhimurium scheme (Larsson et al., 2009) it is not trivial to search for type matches.

The discriminatory powers of the five different loci included in the MLVA analysis were calculated for the 100 isolates and it was found that the loci with highest discriminatory power were STTR5 and STTR6 (Table 2). For all loci, apart from STTR9, there were some strains for which no amplicon was obtained, with the highest number of isolates for STTR10 (Table 2, Table S1). The discriminatory ability of the different MLVA loci corresponded well with previous investigations (Dimovski et al., 2014; Löfström, Hintzmann, Sørensen, & Baggesen, 2015; Wuyts et al., 2013), showing that STTR5, STTR6, and STTR10 were the loci with highest discriminatory index (DI) whereas a lower DI was observed for STTR3 and STTR9. STTR10 (plasmid borne) has in previous investigations been shown to be absent in many strains, thus being less useful for inclusion in the typing scheme. This is similar to our study, where 37% of the strains were found to lack this locus. Variants with absent loci were also found for STTR3, STTR5, and STTR6, in agreement with previous investigations (Wuyts et al., 2013). Moreover, an unusual combination of variants lacking both STTR3 and STTR9 was observed for 7% of the isolates. A high occurrence of variants with missing loci hampers the future use of MLVA and calls for the inclusion of additional loci and/or supplementing with another typing method to obtain the desired discriminatory ability.

**Table 2:** Comparison of results obtained for the five loci included in the multiple‐locus variable‐number of tandem repeats analysis

Locus	No. of variants	No. of isolates where locus is not present	No. of isolates with highest frequency variant	Hunter–Gaston′s index of diversity (95% CI)
STTR5	18	7	17	0.898 (0.876–0.921)
STTR9	5	0	76	0.400 (0.291–0.510)
STTR6	19	4	22	0.895 (0.862–0.927)
STTR10	21	37	37	0.840 (0.778–0.901)
STTR3	6	13	36	0.721 (0.674–0.768)
Total	80	N/A	5	0.994 (0.990–0.998)

N/A, not applicable.

Contrary to other studies, the MLVA types found were evenly distributed over the investigated isolates, with no MLVA group containing more than five isolates. In two previous investigations using a greater number of strains, six MLVA types represented 61% (Arguello et al., 2014) and 64% (Wuyts et al., 2013), respectively, of the total number of isolates, thus giving rise to a question about the applicability of MLVA for monitoring purposes. However, when joining SLVs into groups, the 80 MLVA types for the 100 Bulgarian isolates were merged into 60 groups. This criterion has often been applied to find epidemiologically related strains in outbreak investigations (Torpdahl, Sorensen, Lindstedt, & Nielsen, 2007). More recently it has been proposed to join MLVA types with the identical alleles for STTR3 and STTR9, but with a one allele difference in the more rapidly changing loci STTR5, STTR6 and/or STTR10 together to take into account the difference in stability over time for the different loci (Dimovski et al., 2014).

The AMR patterns of the strains were also investigated in this study. Among the 100 isolates tested for antimicrobial resistance, 83 were resistant to at least one antimicrobial agent (Tables S1 and S2). The most common resistance patterns were ASSuT (*n* = 11), ACSSuT (*n* = 6), Su (*n* = 6), ACSu (*n* = 5), and ACSSu (*n* = 5). Seventeen of the strains were susceptible to all agents. Resistance to ≥4 antimicrobial agents was present in 44 of the 100 isolates. Notably, no isolates were resistant to cephalosporins (cefotaxime, ceftazidime, cefuroxime axetil, or cephalothin), amikacin, or trimethoprim, whereas resistance to carbenicillin, gentamicin, and fluoroquinolones (ciprofloxacin) was low. Resistance was highest to ampicillin, streptomycin, sulfonamides, and tetracycline. For some compounds there was a significant difference in resistance levels between clinical isolates and the food and veterinary isolates. Thus, for gentamicin (*p* = 0.0017), sulfonamides (*p* = 0.045), and carbenicillin (*p* = 0.001) resistance levels were higher among the human isolates, whereas for nalidixic acid (*p* = 0.027) there was more resistance among the food and veterinary isolates. Most resistance was recorded to ampicillin, chloramphenicol, streptomycin, sulfonamides, and tetracycline, which are the resistance factors of the typical DT104 clone. This is similar to previously reported studies. Similar to others, it was found that among ceftazidime‐resistant isolates, the most frequent pattern was resistance to ampicillin, streptomycin, tetracycline, sulfonamide, and ceftazidime (ASTSuCaz) (Adhikari et al., 2010). No isolates were resistant to the critical compounds cephalosporin, and resistance was low to another critical group of compounds, the fluoroquinolones. For gentamicin, sulfonamides, carbenicillin, and nalidixic acid there was a significant difference in resistance levels between clinical isolates and the food and veterinary isolates. It remains to be determined whether this reflects differences in usage, a methodological difference or other factors. However, carbenicillin is mainly used in human medicine, which may explain the higher resistance among human isolates, whereas quinolones are widely used in veterinary practice, which may explain the higher resistance levels to nalidixic acid among the food and veterinary isolates.

The number of MLVA profiles identified in this study reflects the varying origins of the isolates and the discriminatory power of the method, which can explain the differences compared with previously published data. However, our results are in line with other studies performed to obtain background knowledge about the variability in MLVA among isolates in specific countries (Almeida, Medeiros, Kich, & Falcão, 2016; Hoelzer et al., 2010; Laorden et al., 2010; Wuyts et al., 2013). Also AMR analysis had a high discriminatory power and enabled differentiation of the tested strains in 34 groups. Moreover, there was an overlap between MLVA and AMR groups yielding even higher discrimination (Figure 1, Table S1). However, isolates in this study were selected to be diverse, and the discriminatory ability might therefore not reflect the true value when used for continuous monitoring and further studies are needed to evaluate this.

During recent years WGS has been introduced as a novel typing tool used by, for example, public health laboratories (Ronholm et al., 2016). The use of WGS can overcome some of the obstacles with MLVA, such as a low discriminatory power observed for some serotypes and the challenge with missing loci. It has been shown that WGS has a higher discriminatory power compared with MLVA, as it includes a larger part of the genome in the analysis. Moreover, a good correlation has been found between MLVA and WGS, where a MLVA type is split into different WGS types (Phillips et al., 2016; Wuyts et al., 2015). However, MLVA is a fast screening tool and is cheaper to use compared to WGS.

In conclusion, the results obtained in this study confirmed the usefulness of MLVA in differentiation of *S. *Typhimurium and have demonstrated how new molecular strategies may be used to supplement conventional methods to enable an accurate and rapid comparison of isolates of human and veterinary origin. Furthermore, it was shown that Bulgarian isolates differentiates greatly in the MLVA types found and that the occurrence of multiresistant strains is common among Bulgarian *S. *Typhimurium isolates both from human, food, and veterinary sources. When joining MLVA SLVs together a large cluster of strains from human, food, and veterinary sources was observed, potentially indicating a transfer of *Salmonella* from food and animal sources to humans. However, further studies are needed to investigate this and to evaluate the usefulness of MLVA in outbreak investigations and long‐term monitoring of *Salmonella* in Bulgaria.

## 3 MATERIAL AND METHODS

### 3.1 *Salmonella* isolates

Fifty *S. *Typhimurium isolates recovered from animals and food during the period 2006‐2012 were obtained from the National Reference Laboratory “*Salmonella*,* Campylobacter* and antimicrobial resistance”, National Diagnostic and Research Veterinary Institute, Sofia, Bulgaria and 50 *S. *Typhimurium isolated from humans from the National Reference Laboratory (NRL) of Enteric Pathogens, National Center of Infections and Parasitic Diseases, Sofia, Bulgaria. Isolates were randomly selected from the total number of *Salmonella* isolates. Veterinary and food isolates represented about 35% and human isolates 19% of the total number of *Salmonella* isolates for the period 2006–2012. Isolates originated from human (*n* = 50), food (*n* = 39), and veterinary (*n* = 11) sources (Table S1) and were identified by biochemical methods (API^®^ 20E, BioMerieux, France) and serotyping. Serotypes were determined according to the White–Kauffmann–Le Minor scheme (Grimont & Weill, 2007) using Salmonella O‐ and H‐ antisera (Bulbio, Bulgaria; SIFIN, Germany; and SSI, Copenhagen, Denmark).

### 3.2 Antimicrobial resistance determination

Antimicrobial resistance profiles were determined with the following antimicrobial agents: ampicillin—A (10 μg), cefotaxime—CTX (30 μg), ceftazidime—CAZ (30 μg), chloramphenicol—C (30 μg), ciprofloxacin—CP (5 μg), gentamicin—G (10 μg), nalidixic acid—Na (30 μg), streptomycin—S (10 μg), sulfonamides—Su (300 μg), tetracycline—T (30 μg), trimethoprim—Tm (5 μg), carbenicillin—Cb (100 μg), cefuroxime axetil—Cx (30 μg), cephalothin—Cf (30 μg), and amikacin—Ak (30 μg)using the Bauer–Kirby disk diffusion method (Bauer, Kirby, Sherris, & Turck, 1966). Clinical and Laboratory Standards Institute (CLSI) criteria were applied for interpretation of antibiograms (CLSI, 2010).

### 3.3 MLVA typing

The procedure suggested by ECDC was applied (ECDC 2011), using primers as previously described (Larsson et al., 2009; Lindstedt, Vardund, Aas, & Kapperud, 2004). Capillary electrophoresis was performed on a CEQ 8,000 Genetic Analysis System (Beckman Coulter, Fullerton, CA, USA).

### 3.4 Data analysis

MLVA allele numbers were analyzed with BioNumerics v. 7.1 (Applied Maths, Sint‐Martens Latem, Belgium) as character values, and MSTs were constructed using categorical coefficients and the Ward algorithm (Ward, Hastie, Barry, Elith, & Leathwick, 2009). The following priority roles were used to create networks: (1) Maximum number of N‐locus variants (*N* = 1) Weight: 10,000 and (2) Maximum number of N‐locus variants (*N* = 2) Weight: 10.

Discriminatory power and its confidence interval were calculated using Simpson's and Shannon′s indices of diversity, as previously described (Hunter & Gaston, 1988; Magurran, 1988) using Bionumerics v 7.1 and the V‐DICE diversity calculator from Public Health England available at: http://www.hpa-bioinformatics.org.uk/cgi-bin/DICI/DICI.pl. The Shannon–Weiner diversity index describes how evenly the subtypes are distributed in the sampled population.

Differences between levels of antimicrobial resistance between different groups of isolates (human, food or veterinary origin) were determined using χ^*2*^ tests. A *p* value ≤0.05 was considered statistically significant.

## CONFLICT OF INTEREST

The authors declare no potential conflicts of interest.
