# Supplementary material for: Use of multiple‐locus variable‐number of tandem repeats analysis (MLVA) to investigate genetic diversity of Salmonella enterica subsp. enterica serovar Typhimurium isolates from human, food, and veterinary sources
Source: Microbiologyopen. 2017 Aug 23;7(1):e00528. doi: 10.1002/mbo3.528 (PMC5822324; doi:10.1002/mbo3.528)
Supplement: Supplementary file 1 [file MBO3-7-na-s001.docx]

**Supplementary tables Mateva et al. Use of multiple-locus variable-number tandem-repeats analysis (MLVA) to investigate genetic diversity of *Salmonella enterica* subsp. *enterica* serovar Typhimurium isolates from human, food and veterinary sources**

**Supplementary Table 1**. List of *Salmonella* ser. Typhimurium strains included in the study and results on multi locus variable number of tandem repeat analysis (MLVA) and antimicrobial resistance (AMR) patterns.

| **Strain no.** | **Source (human/food/veterinary)** | **Source**  **(human/pork/beef/**  **poultry/unknown/other)** | **Year of isolation** | **MLVA type^a^** | **AMR pattern^b^** |
| --- | --- | --- | --- | --- | --- |
| 40 | Food | Beef | 2006 | 1-11-9-7-NA | Sensitive |
| 32 | Food | Unknown | 2006 | 1-9-NA-NA-111 | CpNa |
| 22 | Food | Unknown | 2006 | 2-18-8-14-212 | ACpNa |
| 26 | Veterinary | Eggs | 2006 | 3-12-11-21-311 | Su |
| 27 | Veterinary | Eggs | 2006 | 3-12-11-21-311 | Su |
| 25 | Food | Poultry | 2006 | 3-12-11-21-311 | Su |
| 33 | Food | Beef | 2006 | 3-12-13-21-311 | Su |
| 5 | Food | Pig | 2006 | 3-14-14-13-311 | ACSSuT |
| 13 | Food | Poultry | 2006 | 3-14-14-13-311 | ACSSu |
| 21 | Food | Pig | 2006 | 3-7-15-25-311 | ACNaSSuT |
| 17 | Food | Pig | 2006 | 5-19-10-NA-211 | ACCpSSuT |
| 42 | Food | Poultry | 2007 | 2-12-3-21-212 | CpNa |
| 1615 | Human | Human | 2007 | 3-12-10-12-211 | ACbT |
| 2230 | Human | Human | 2008 | 2-23-10-NA-111 | ASSuT |
| 2294 | Human | Human | 2008 | 2-23-12-22-111 | ASuT |
| 2173 | Human | Human | 2008 | 2-23-12-NA-111 | Sensitive |
| 2168 | Human | Human | 2008 | 2-23-NA-16-111 | Sensitive |
| 2175 | Human | Human | 2008 | 2-23-NA-NA-111 | Sensitive |
| 1844 | Human | Human | 2008 | 2-NA-18-12-NA | Cp |
| 1847 | Human | Human | 2008 | 3-10-11-12-NA | Sensitive |
| 2268 | Human | Human | 2008 | 3-11-10-2-211 | ACGSSuT |
| 1889 | Human | Human | 2008 | 3-11-9-11-211 | ACGSSuT |
| 1960 | Human | Human | 2008 | 3-11-9-22-211 | ACCbGT |
| 1941 | Human | Human | 2008 | 3-11-9-8-NA | GSuT |
| 1900 | Human | Human | 2008 | 3-11-9-NA-211 | ACGSSuT |
| 2378 | Human | Human | 2008 | 3-11-9-NA-211 | ACCbGST |
| 2379 | Human | Human | 2008 | 3-11-9-NA-211 | ACbT |
| 2392 | Human | Human | 2008 | 3-11-9-NA-211 | ACCbGST |
| 2293 | Human | Human | 2008 | 3-12-10-NA-NA | ASSuT |
| 120 | Food | Unknown | 2008 | 3-12-9-NA-211 | AT |
| 2270 | Human | Human | 2008 | 3-12-9-NA-211 | ASSu |
| 2224 | Human | Human | 2008 | 3-13-13-23-311 | ACSu |
| 2273 | Human | Human | 2008 | 3-13-13-NA-211 | ASSuT |
| 2282 | Human | Human | 2008 | 3-13-14-NA-211 | ASSuT |
| 94 | Food | Unknown | 2008 | 3-13-15-19-311 | NaSu |
| 73 | Food | Unknown | 2008 | 3-13-18-20-311 | ACSSu |
| 1853 | Human | Human | 2008 | 3-13-5-11-311 | ACCbT |
| 1956 | Human | Human | 2008 | 3-14-12-22-311 | ACSu |
| 2377 | Human | Human | 2008 | 3-14-9-NA-311 | ACCbT |
| 2205 | Human | Human | 2008 | 3-22-6-NA-311 | ACSSu |
| 1846 | Human | Human | 2008 | 3-NA-10-27-NA | Cp |
| 1997 | Human | Human | 2008 | 3-NA-12-23-NA | ACSu |
| 1854 | Human | Human | 2008 | 3-NA-5-11-NA | ACCbT |
| 2807 | Human | Human | 2009 | 2-NA-4-NA-NA | Sensitive |
| 237 | Food | Pig | 2009 | 3-11-10-21-211 | AST |
| 2770 | Human | Human | 2009 | 3-11-9-11-211 | ACGT |
| 2727 | Human | Human | 2009 | 3-11-9-1-211 | ACGT |
| 2729 | Human | Human | 2009 | 3-11-9-NA-211 | ACGST |
| 2811 | Human | Human | 2009 | 3-12-11-8-NA | Sensitive |
| 2829 | Human | Human | 2009 | 3-12-15-21-311 | ACSu |
| 2777 | Human | Human | 2009 | 3-13-12-21-311 | Sensitive |
| 2792 | Human | Human | 2009 | 3-14-9-21-311 | ACSuT |
| 2793 | Human | Human | 2009 | 3-14-9-21-311 | ACSuT |
| 2800 | Human | Human | 2009 | 3-16-17-25-NA | ACCpNaT |
| 2733 | Human | Human | 2009 | 3-NA-12-21-NA | T |
| 2813 | Human | Human | 2009 | 3-NA-4-11-NA | Sensitive |
| 246 | Food | Poultry | 2010 | 1-8-8-16-112 | CpNaSSu |
| 251 | Veterinary | Poultry | 2010 | 2-20-18-12-212 | Sensitive |
| 247 | Food | Poultry | 2010 | 2-21-NA-NA-111 | Sensitive |
| 258 | Food | Unknown | 2010 | 3-11-13-NA-211 | ASSuT |
| 238 | Food | Unknown | 2010 | 3-11-17-16-311 | ACST |
| 239 | Food | Unknown | 2010 | 3-11-17-16-311 | ACST |
| 244 | Food | Unknown | 2010 | 3-13-10-9-211 | AST |
| 240 | Food | Unknown | 2010 | 3-13-10-NA-211 | ASSuT |
| 252 | Veterinary | Pig | 2010 | 3-13-12-NA-211 | ACSSuT |
| 242 | Food | Pig | 2010 | 3-14-14-15-311 | ACSSu |
| 241 | Food | Poultry | 2010 | 3-14-14-15-311 | ACSSu |
| 250 | Veterinary | Pig | 2010 | 3-14-16-35-311 | ACCpNaSSu |
| 124 | Human | Human | 2010 | 3-15-10-NA-311 | A |
| 126 | Human | Human | 2010 | 3-17-10-8-311 | ASu |
| 118 | Human | Human | 2010 | 3-17-10-NA-311 | ACb |
| 125 | Human | Human | 2010 | 3-17-10-NA-311 | A |
| 127 | Human | Human | 2010 | 3-17-10-NA-311 | A |
| 245 | Food | Unknown | 2010 | 4-12-8-NA-211 | Cp |
| 128 | Human | Human | 2010 | 4-14-11-8-211 | ACCbT |
| 243 | Food | Pig | 2010 | 4-14-8-12-211 | AST |
| 196 | Veterinary | Feed | 2011 | 2-19-9-7-311 | Sensitive |
| 197 | Veterinary | Feed | 2011 | 2-20-9-7-212 | Su |
| 88 | Human | Human | 2011 | 3-11-6-12-211 | Sensitive |
| 255 | Veterinary | Poultry | 2011 | 3-12-22-13-311 | Sensitive |
| 248 | Food | Unknown | 2011 | 3-14-7-15-211 | SSuT |
| 188 | Human | Human | 2011 | 3-15-17-14-311 | ACSu |
| 89 | Human | Human | 2011 | 3-15-8-13-311 | ASu |
| 254 | Veterinary | Pig | 2011 | 3-16-11-NA-211 | AT |
| 256 | Veterinary | Pig | 2011 | 3-17-10-NA-211 | AT |
| 288 | Food | Eggs | 2012 | 2-20-9-7-212 | Sensitive |
| 289 | Food | Eggs | 2012 | 2-20-9-7-212 | Sensitive |
| 307 | Food | Pig | 2012 | 3-12-10-NA-211 | ASSuT |
| 315 | Food | Pig | 2012 | 3-12-10-NA-211 | ACSSuT |
| 259 | Food | Pig | 2012 | 3-12-9-NA-211 | ASSuT |
| 272 | Veterinary | Pig | 2012 | 3-13-10-NA-211 | ASSuT |
| 67 | Human | Human | 2012 | 3-13-9-14-311 | Sensitive |
| 282 | Food | Unknown | 2012 | 3-14-10-13-211 | ASSuT |
| 283 | Food | Pig | 2012 | 3-14-10-NA-211 | ASSuT |
| 309 | Food | Unknown | 2012 | 3-15-13-NA-311 | A |
| 311 | Food | Unknown | 2012 | 3-7-1-16-311 | ACSSuT |
| 312 | Food | Pig | 2012 | 3-7-16-17-311 | ACSSuT |
| 304 | Food | Unknown | 2012 | 3-7-6-17-311 | ACSSuT |
| 260 | Veterinary | Poultry | 2012 | 4-14-4-NA-211 | Su |
| 269 | Food | Unknown | 2012 | 4-14-4-NA-211 | T |

^a^MLVA pattern given in the following order: STTR9, STTR5, STTR6, STTR10, STTR3

^b^A: ampicillin, Ctx: cefotaxime, Caz: ceftazidime, C: chloramphenicol, , Cp: ciprofloxacin, G: gentamicin, Na: nalidixic acid, S: streptomycin, Su: sulfonamides, T: tetracycline, Tm: trimethoprim, Cb: carbenicillin, Cx: cefuroxime axetyl, Cf: cephalothin, Ak: amikacin

**Supplementary Table 2**.Results for the antimicrobial resistance (AMR) of the 100 Bulgarian *Salmonella* serovar Typhimurium strains.

| Sample type  (no. of isolates) | No. of isolates (%) resistant towards the indicated antimicrobial agent^a^ | | | | | | | | | | | | | | | No. of isolates (%) resistant towards  indicated no. of antimicrobial agents | | |
| --- | --- | --- | --- | --- | --- | --- | --- | --- | --- | --- | --- | --- | --- | --- | --- | --- | --- | --- |
|  | **A** | **C** | **Caz** | **Cp** | **Ctx** | **G** | **Na** | **S** | **Su** | **T** | **Tm** | **Cb** | **Cx** | **Cf** | **Ak** | **0** | **1-3** | **4-6** |
| Food + veterinary  (50) | 30  (60) | 15  (30) | 0  (0) | 2  (4) | 0  (0) | 0  (0) | 7  (14) | 27  (54) | 29  (58) | 25  (50) | 0  (0) | 0  (0) | 0  (0) | 0  (0) | 0  (0) | 8  (16) | 20  (40) | 22  (44) |
| Human  (50) | 36  (72) | 22  (44) | 0  (0) | 1  (2) | 0  (0) | 9  (18) | 1  (2) | 12  (24) | 19  (38) | 25  (50) | 0  (0) | 10  (20) | 0  (0) | 0  (0) | 0  (0) | 12  (24) | 18  (36) | 20  (40) |
| Total  (100) | 66  (66) | 37  (37) | 0  (0) | 3  (3) | 0  (0) | 9  (9) | 8  (8) | 39  (39) | 48  (48) | 50  (50) | 0  (0) | 10  (10) | 0  (0) | 0  (0) | 0  (0) | 20  (20) | 38  (38) | 42  (42) |

^a^ A: ampicillin, Ctx: cefotaxime, Caz: ceftazidime, C: chloramphenicol, ,Cp: ciprofloxacin, G: gentamicin, Na: nalidixic acid, S: streptomycin, Su: sulfonamides, T: tetracycline, Tm: trimethoprim, Cb: carbenicillin, Cx: cefuroximeaxetyl, Cf: cephalothin, Ak: amikacin
